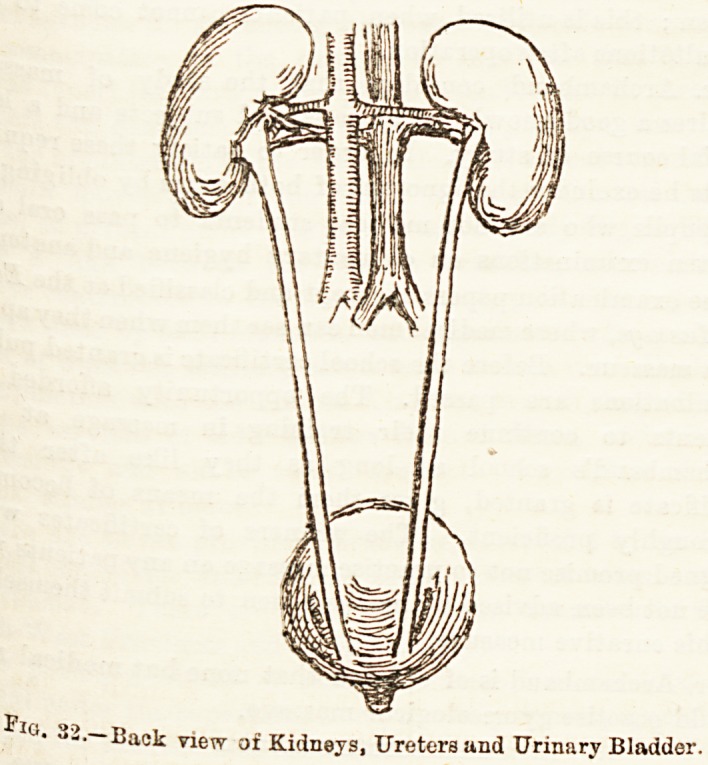# The Hospital Nursing Supplement

**Published:** 1895-06-29

**Authors:** 


### "The Hospital" Nursing Mirror.

Being the Extra Nursing Supplement of "The Hospital" Newspaper.

[Contributions for this Supplement should be addressed to the Editor, The Hospital, 428, Strand, London, W.C., and should have the word "Nursing" plainly written in left-hand top corner of the envelope.]

### News from the Nursing World.

### THE NIGHTINGALE TRAINING SCHOOL.

A brilliant reception was given on 20th inst. at the Nightingale Home, St. Thomas's Hospital, and a large number of past and present members of the medical staff and a great many past and present Nightingale probationers spent a most enjoyable afternoon. The statue of Miss Nightingale has an honoured place in the great dining hall, where the guests were received by Mr. Bonham Carter, Secretary to the Nightingale Fund, Mrs. Bonham Carter, and the Matron, Miss Gordon. Taking advantage of the glorious weather, the visitors afterwards sat out in the picturesque quadrangle, where the band was playing, or wandered along the fine terrace bordering the Thames. The wards also attracted attention, and "old Nightingales" were glad to congratulate the present generation of nurses on the improved accommodation provided for them nowadays. Formerly they moved out of the pleasant separate bedrooms at the Home at the end of their probationary year into cubicles divided only by low partitions. During the reign of the present matron these have been altered, and each nurse has a room whose walls reach the ceiling. A recent welcome addition has been made to the nurses' quarters of several prettily fitted and furnished bedrooms. If Miss Nightingale had been able to attend in person last week's pleasant entertainment, in the success of which she undoubtedly takes a warm interest, she must have felt proud of the distinguished women, many of them now heads of hospitals, who flocked to visit their old training school at "the Nightingale Home."

### PRELIMINARY TRAINING.

Tredegar House, 99, Bow Road, E., is detached and substantial-looking, and possesses a very large garden. It will be occupied in a few days by twenty pupil-probationers, for whom the committee of the London Hospital have provided most excellent accommodation. There is a pleasant dining-room, a study, with small separate tables, and a large lecture or class room. The bed-rooms are admirably furnished and very comfortable, whilst the sanitary arrangements, as well as the various offices, leave nothing to be desired. Tredegar House appears to afford every facility for the practical preliminary training of the pupil-probationers for whom it has been fitted up. Miss Lückes, matron of the London Hospital, is to be congratulated on the initial success of her orginal scheme.

### "THE HOSPITAL" CONVALESCENT FUND.

"I am sure you will all be pleased to hear how much better I feel for my visit to the sea," writes a nurse, "I am really quite set up again and fit for work. I hope by and by to be able to send a donation to The Hospital Convalescent Fund." After a severe illness this writer, a widow, found herself without means and without strength to resume nursing. Alone in lodgings, insufficient and unsuitable food only attainable, her condition grew daily worse. Hearing of The Hospital Convalescent Fund she applied to the hon. secretaries, and as soon as they received a letter from her doctor recommending an immediate change of air and scene, they arranged for a three weeks' visit to a convalescent seaside home. The home was one to which the patient herself was anxious to be sent, and the results of her stay there have been most satisfactory. The knowledge that the combined efforts of nurses founded the little fund from which they are helped appears to add to the pleasure and gratitude of those who benefit by it. Subscriptions to allow of further help to weary and convalescent nurses who are unable to meet the expenses of enforced holidays, are earnestly asked for. Donations and subscriptions should be sent to the hon. secretaries, The Hospital Convalescent Fund, 428, Strand, London.

### AN UNFORESEEN CONCLUSION.

"Well, Jane, how do you like the new doctor?" asked the district nurse of a sick cottager. "He seems a clever sort of young man," returned the latter, "and he's heartened me up above a bit!" The nurse looked rather puzzled. "I was afraid I should have found you rather down, Jane, for I heard the doctor had been saying you ought to have an operation if you wanted ever to get well." "So he did," returned the sick woman, "so he did! And I asked him whether he thought he should be able to do it himself. He says, briskly, 'Oh! yes, certainly.' He should like to have another doctor to look on, he said, but he could manage it all right himself. So when he'd spoken I up and thanked him. 'Well, sir,' I says, 'you've eased my mind wonderful, and now I needn't trouble you to come again. I thought there wasn't much amiss with me, and you see I'm right, after all. It stands to reason that a young man like you wouldn't do anything as wasn't quite easy, so I'll just be left alone for the present!'"

### SLIGHTED RECOMMENDATIONS.

The most superficial newspaper-reader can hardly fail to realise the varied uses to which the name of "the Local Government Board" adapts itself. For all abuses which come to light in connection with the nursing of sick paupers the Board is held directly responsible by the public, yet it can only "recommend" and "advise"! For example, the appointment of a trained head nurse in an infirmary may be suggested, and the Guardians nevertheless decide that some woman without a certificate, and totally unqualified, is the suitable candidate. Long and tedious correspondence ensues, the public blaming the Local Government Board for not using authority which it does not possess. In 1892 a circular issued by the Board called attention to the important subject of trained nursing in workhouses, yet when this communication was again distributed in the current year the discussions which arose showed how little previous attention had been paid to it. The power possessed by Guardians to appoint their own district nurses is little appreciated, although on February 1st, 1892, a circular, headed "District Nurse," was issued to Boards of Guardians all over the country, not only sanctioning the employment of such persons as duly appointed officers, but laying down the principle that they "should have had thorough practical training in the nursing of the sick"; also "that special personal qualifications are necessary," and "experience in nursing the sick poor in their own homes." The period to be spent by the nurse in the training school of a hospital or infirmary is laid down at one year, at least, although, it is remarked, a longer period "would seem desirable."

### PROVISION FOR INCURABLE CHILDREN.

Letters signed "Humanitarian" have appeared in several provincial papers headed "National County Homes for Incurable Children." The writer suggests the establishment by Government of county homes for physically afflicted children, each county to have an estate with a church, school, and hospital, fruit gardens, &c. Private philanthropy would be requisitioned to supplement the work which the Government may be induced to undertake. "Humanitarian" suggests that the "helpers" should be volunteers with sufficient means for their own support, and that the friends of the children should pay a weekly sum for the children's maintenance. "Humanitarian" would do well to make himself acquainted with the many excellent convalescent homes and hospitals already existing in most parts of England, where private local charity already provides many little patients with the care and skill which he craves for them. These establishments are often managed by nurses and other competent ladies, who give time, money, skill, and strength to the service of invalid children. More provision for chronic and incurable cases is sorely needed, and "Humanitarian" and his friends might perhaps be content to raise funds to support extra beds or enlarge existing institutions.

### NURSES IN CHELTENHAM.

The annual meeting of the Cheltenham District Nursing Association, held last week, was well attended, the Rev. Canon Hutchinson, R.D., being in the chair. An interesting address on the subject of trained nursing for the poor in their own homes was given by Miss Wallich after the general business of the meeting had been disposed of. During the present year 16,877 visits have been paid to 672 cases, and the maternity branch has done valuable work both for patients and in training pupils under the midwives.

### NURSING BY SISTERHOODS.

The plea that Sister de Sales O'Connell has had "considerable experience," although she is not a trained nurse, is no new argument from the lips of those who imagine that a gentle and pious sister of charity must needs be also a heaven-born nurse. The Lord Bishop of Ardagh appears much displeased at the Local Government Board of Ireland dissenting from his opinion. They venture to insist on the appointment of a qualified trained nurse for the sick poor in Athlone workhouse; and as the Bishop has threatened to withdraw the nuns from the hospital if a lay nurse is appointed, it would seem that no resident nun there has qualified for the nursing as well as religious profession of a Sister. Whilst a higher standard of training is each year demanded of lay nurses, that prevailing in religious communities appears to remain almost stationary. Some "Sisters" are admitted to certain hospitals for comparatively short periods, and, in their insanitary black woollen garments, they move softly about, learning as much as can reasonably be expected of them. They share the theoretical instruction given to ordinary probationers, but not their practical work. After perhaps twelve months spent in close attendance in the wards and going in for certain examinations as to proficiency, one of these "Sisters" may be immediately sent off to take a position of responsibility. If she should be drafted abroad, she may be entirely dependant on native assistants. In giving over any hospital wards to the charge of a religious community, the managers of the institution should make sure that full training under qualified teachers has been received by those to whom they entrust the helpless sick. Kindliness and courtesy, however valuable accessories, are not efficient substitutes for skill and experience.

### TRAINING IN PARIS.

The classes held at three of the hospitals in Paris, for the instruction of nurses and attendants, having become inconveniently overcrowded, a fourth has been added to the list of municipal training schools. It is connected with the Lariboisière Hospital, an institution occupying a central position, and the classes, now in full working order, are attended by mothers and nurses in the neighbourhood. The classes are open to all, and are quite free. Some 119 attendants and nurses are at present going through the course, besides the other pupils. The director of the Lariboisière Hospital has encouraged his staff to avail themselves of this technical education, and it seems likely that a considerable number of the seniors will be found at the end of the scholastic year to have gained certificates. They will thus be better equipped in theoretical and practical knowledge in the future than they have been in the past, and the physicians and surgeons will secure more intelligent assistants, whilst the sick will receive more skilled attendance. The progress made in nursing in England and America has been long in rousing emulation in France, and it will be some time ere a full course of training is acknowledged as essential for sick nurses and attendants.

### SHORT ITEMS.

The Totnes Guardians have decided to advertise for a trained and certificated head nurse for their infirmary.—The Epping District Council are reported to have rescinded their resolution to appoint a certificated nurse.—The Community of the Epiphany have offered to provide a trained district nurse for Truro at £75 per annum.—Although some of the Guardians at Northampton wished to advertise for a head nurse for the workhouse infirmary, a majority voted in favour of the appointment of Miss Fanny Daniels, of Berry Wood Asylum, to the post.

### Elementary Anatomy and Surgery for Nurses.

By W. McAdam Eccles, M.B., M.S., F.R.C.S., Lecturer to Nurses, West London Hospital, &c.

### XXIII.—THE EXCRETORY SYSTEM—THE ABSORPTIVE SYSTEM.

During the various chemical and other changes which take place in the tissues, various waste matters are produced which, if allowed to continue in the circulation, would do harm. Certain organs have therefore been provided whereby these may be eliminated and removed, which are known as the excretory organs. These may be classified as: (1) The kidneys or renal organs, with their ducts, the ureters, and the receptacle for their secretion, the urinary bladder; (2) the sweat glands of the skin; (3) the liver; (4) the intestines; (5) the lungs. Of these only the kidneys and sweat glands will here have to be dealt with, the other organs having been previously described.

The kidneys, two in number, are placed one on either side of the upper lumbar vertebræ at the back of the abdomen. They are each about four inches in length, two and a-half in width, and one and a half in thickness, with a weight of about four and a half ounces. They are somewhat oval in form, with an outer convex and an inner concave border, in fact they are kidney-shaped. They lie embedded in a mass of fat, popularly called suet, and have a capsule or fibrous coat surrounding them. On the inner side, at a spot called the hilum, enters the renal artery, a large branch of the abdominal aorta, and the renal vein which joins the inferior vena cava leaves at the same spot together with the duct of the kidney known as the ureter. (See fig. 32.)

It will be observed that a considerable quantity of blood is constantly passing through the vessels of the renal organ

Fig. 32.—Back view of Kidneys, Ureters and Urinary Bladder.

from which the waste products are removed and water abstracted to form the excretion called urine, which fluid flows down the ureters and collects in the hollow muscular organ termed the bladder. This lies in the pelvis and lower part of the abdomen close behind the symphysis pubis. It is oval or conical in shape, and has three openings, two of which, the terminations of the ureters, are at the lower and posterior part, and the third is the commencement of the channel called the urethra, through which the urine is voided. About the beginning of this duct, that is at the neck of the bladder, is a collection of unstriped muscular fibres arranged circularly, and constituting a sphincter muscle which, when contracted, prevents the outflow of urine.

The sweat glands are extremely numerous, especially so in certain regions such as the armpits, nose, and forehead. They are situated in the skin, and consist of a much-coiled tube with a duct, which in passing through the epidermis is twisted like a corkscrew. They are well supplied with blood.

### The Absorptive System.

This consists of a system of tubes and filtering glands. The tubes are called lymphatic capillaries and vessels, and these, passing through lymphatic glands, unite to form two lymphatic trunks, the larger of which is called the thoracic duct. These trunks themselves empty, one on either side, into the junction of the internal jugular with the subclavian vein at the root of the neck. All the tissues of the body are permeated by lymphatic capillaries. They serve the double function of receiving a great part of the digested substances called chyle from the alimentary tract, and of absorbing excess of fluid from other tissues.

The lymphatics of the digestive system are very abundant in the walls of the intestine, and joining together form larger vessels known as lacteals. These after passing their contents through the meshes of lymphatic glands situated in the mesentery for the most part discharge them into a dilated portion of the thoracic duct known as the receptaculum chylæ. From this receptacle the duct is continued up through the diaphragm and thorax to empty as above stated. In its course it contains many valves. The lymphatic vessels of the trunk and limbs are commonly divided into a superficial and deep set. They arise in the spaces between the constituents of a tissue, and after a certain longer or shorter course, pass into lymphatic glands where their contents are filtered. The position of the chief of these glands it is important to know, since during the process of cleansing the lymph they are liable at times to become inflamed. The lymphatics of the scalp, face, ear, mouth, and its contained structures pass into a number of glands situated below the jaw and in the upper part of the neck laterally.

In the axilla are three sets of glands, the first along the axillary vessels into which pass the lymphatics of the upper extremity, except those of the inner side of the hand and forearm, which convey their lymph to a single gland placed a little above the internal condyle of the humerus—the supracondylar gland. The second set are a series which run down in front of the posterior fold of the axilla, and derive their lymph vessels from the shoulder and upper part of the back. The third is a set placed behind the anterior fold of the axilla, to which run the lymphatics of the chest wall, including most of those of the breast.

In the groin are two groups of glands, one of which, placed nearly horizontally above Poupart's ligament, receives vessels from the abdominal wall, the lower part of the back, including the buttocks, and from the perineum, or region about the anus. The other set is vertical below the ligament, and into the glands composing it empty the lymph channels of the lower extremity, excluding those from the heel and back of the leg, which pass into some glands situated in the popliteal space behind the knee-joint.

### Royal National Pension Fund.

Such a large number of letters have been addressed to the Secretary of the Royal National Pension Fund for Nurses, inquiring about the uniform to be worn at the reception at Marlborough House, that he asks us to insert an answer: "Indoor uniform is certainly recommended for all who generally have it. District nurses, of whose costume bonnets form an essential part, can wear them in place of caps if they like, but cloaks are better omitted in all cases."

### Nursing in Ireland.

### BARRINGTON'S HOSPITAL, LIMERICK.

Barrington's Hospital and City of Limerick Infirmary is situated in the oldest portion of the city of Limerick, that known as "Irishtown." The hospital is a rather picturesque building of cut grey stone, the cathedral, castle, and treaty stone being in its immediate vicinity.

Founded in 1829 on the site of the old Main Guard House by Sir Joseph Barrington, Bart., and his sons, the hospital was opened for the reception of patients on November 5th, 1831, doing good service during the cholera outbreak in the following year, and steadily adding to its record of usefulness up to the present time.

Alterations and improvements have, of course, been made from time to time, but the main building still remains much as it was originally designed, and in some respects, such as insufficient width of corridors and staircases, &c., compares unfavourably with more modern structures. The committee of management have carried out within the last few years, and still contemplate, so many other important improvements, that extensive alterations in the main building cannot be thought of at present.

The conversion of the adjoining old city dispensary rooms, three in number, into wards for private cases is one of the principal changes lately accomplished, and the railing in and laying out of a recreation ground for convalescent patients as a memorial of the late Sir Croker Barrington, Bart., must also be recorded. Still more important would appear to be the contemplated improvement in the nurses' quarters, for which funds are being raised, principally by means of a fancy fair and fête, similar to those which have this year proved so successful in Dublin and Cork. It is to be hoped a sufficient sum may be realised, as at present the accommodation for the nursing staff is most unsatisfactory.

Barrington's Hospital no longer draws its nurses, as it formerly did, from the City of Dublin Nursing Institution, but trains its own probationers, maintaining a sufficient staff for the hospital itself, and supplying private cases in the city and county.

Over 500 intern patients are annually admitted to the hospital, while about 3,000 extern cases are treated every year in the dispensary; accidents, according to the original design of the founders, being preferred to medical cases. Those responsible for the management of the hospital seem fully sensible of the need of constant progress and improvement, so it may be confidently hoped that "Barrington's" record of usefulness in the past may be worthily maintained in the future.

### Royal British Nurses' Association.

The quarterly meeting of the General Council of the Royal British Nurses' Association will be held on Friday, July 12th, at five p.m., at the offices of the association, 17, Old Cavendish Street.

### Appointments.

[It is requested that successful candidates will send a copy of their applications and testimonials, with date of election, to The Editor, The Lodge, Porchester Square, W ]

Repton School Sanatorium.—Miss M. Turner has been appointed Matron of this sanatorium. She was trained at the West Kent General Hospital, Maidstone, and afterwards worked on the private staff there. Miss Turner held the post of lady superintendent of the County of Cornwall Trained Nurses' Home for two and a half years. She has good testimonials, and we wish her every happiness in her new work.

### A Paris School of Massage.

By Madame William Vignal.

The school of massage recently organised by Dr. Archambaud at 15, Rue Mechaux, assists a class of indigent patients who could with difficulty be treated elsewhere. It likewise provides those desirous of practising massage with the means of becoming proficient in their profession.

The school is situated in a healthy part of Paris, sufficiently distant from the centre to be surrounded by gardens, and yet easy of access to patients and pupils. There are two massage-rooms, one for men and one for women patients, and a consultation-room where Dr. Archambaud examines the patients and diagnoses their cases. These are written into a book of observations containing at the present time 250 cases. The consultations are held three times a week; on an average thirty or forty patients are treated at them by the pupils under the direction of Dr. Archambaud. Masseuses treat the women, masseurs the men; and each pupil has his or her patient, who is always treated by the same pupil. Lectures given by Dr. Archambaud complete the technical teaching provided by the consultations; in them he sums up in a masterly way what to do and how to do it, and likewise, what is equally important, what not to do.

The pupils pay an entrance fee of £2 10s. which entitles them to attend the lectures and consultations as long as they may desire to do so.

Dr. Archambaud practises asepsis, and the operation-room has oil-painted walls which can be easily washed. The operations performed at the *Ecole de Massage* are for club-foot, knock-knees, and similar affections; the other operations performed are bloodless. By the side of the operation-room is a cheerful well-furnished bedroom looking on to the garden; this is utilised when patients cannot come to the consultations after operation.

Dr. Archambaud considers that the study of massage requires a good knowledge of collateral subjects and a long special course of study. In order to satisfy these requirements he excludes the ignorant of both sexes by obliging all his pupils who are not medical students to pass oral and written examinations on elementary hygiene and anatomy. These examination papers are kept and classified at the *Ecole de Massage*, where medical men can see them when they apply for a masseur. Before the school certificate is granted public examinations are passed. The opportunity afforded to students to continue their training in massage at Dr. Archambaud's school as long as they like after their certificate is granted, gives them the means of becoming thoroughly proficient. The winners of certificates write a signed promise not to practise massage on any patients who have not been advised by medical men to submit themselves to this curative measure.

Dr. Archambaud is of opinion that none but medical men should practise gynæcological massage.

### Nurses at Bradford Infirmary.

Through the kindness of Mr. C. W. Lupton, chairman of the House Committee, and Mrs. Lupton, the nurses of the Bradford Infirmary spent two most enjoyable days at their residence near Harrogate. One-half of the nursing staff went on the 11th inst., and the other half in the week following. The weather was on both occasions most propitious, luncheon, tea, and refreshments being partaken of on the lawn. Boating on the lake, tennis, cricket, bowls, and short excursions to places of interest were included in the proceedings, the host and hostess being indefatigable in their exertions to make their guests happy. The pleasures of the days will linger long in the memories of all those who participated therein, and the entertainment is one which might be advantageously imitated for the benefit of other infirmary nurses.

### Lady Dufferin's Fund.

To bring medical aid within the reach of all the women of India is a herculean enterprise such as might well daunt the courage and stagger the faith of its promoters, were they not stimulated by the hopeless and desperate need of those they succour. The extent alone of the female population—130 million -lies on the mind with a weight before which the imagination fails. And when to this is added the incessant and bewildering variety of race, creed, and language, each with its own tangle of long-established tradition and custom, and its rigid separation of classes, the whole meeting only in a dogged prejudice against change, some glimmering can be obtained of the overwhelming impediments to the spread of this work.

Missionary zeal first penetrated to the homes of the Indian ladies by means of trained nurses and female medical missionaries, and ascertained that a cordial response was in effect ready for any widespread effort which might hereafter be possible on behalf of the women of India. The impulse to extend the sphere of work and grapple comprehensively with the manifold miseries of womankind in India came in the end from the source best qualified to initiate reform and gain public confidence in that vast empire of conflicting claims, viz., from the Queen-Empress herself. Stimulated by the encouragement and definite recommendation of Her Majesty, Lady Dufferin made her first beginning just ten years ago in raising the Fund which bears her name. From the very first the twofold design of affording medical relief and medical education have gone side by side together. To teach reverence for the laws of health to a people trained to regard loss of life with apathy and indifference, was held no less important than to bring them the comfort of medical and surgical aid in sickness. And the movement, by supplying recognised and attractive careers for women workers, has unexpectedly as it were, developed into one of the most successful agencies for advancing the cause of female education and emancipation in the empire, so much so that in the Madras report for 1894 a complaint is made that in certain quarters this has come to be considered its principal aim.

In the organisation of the Association the actual control of the main movements of the Fund is by the Central Committee, at present under the presidency of Lady Elgin. This forms a link between the distant branches, administers moneys contributed to the Central Fund, publishes reports of work done by local branches, and superintends the preparation of works on sanitary subjects for Zenana use. It also, in conjunction with the United Kingdom branch, negotiates for the services of lady doctors from Europe, and offers scholarships and aid to native students to complete their medical studies at home.

There are eleven provincial branches, controlled by an influential committee, and to these local or district committees are attached. There are 22 of these local branches in the North-West Provinces and 12 in Bombay.

The principal object of each committee is to establish a hospital under the superintendence of a lady doctor, specially arranged for the treatment of "Purda" women, restricted as they are by caste and prejudice from obtaining medical aid elsewhere. Only a few, however, of these local branches have as yet been able to build and support a female hospital, and of these only two or three have been able to afford the salary of a fully qualified lady doctor of the first grade holding a European diploma. Assistant lady surgeons, trained in India, form a second grade, and are much in request; while hospital assistants, provided, it may be presumed, with a very modest measure of preliminary training, form the third grade, and help to eke out the staff. Out of a total of 62 ladies working in connection with the Fund in India, 12 are doctors of the first grade, and 50 are assistant surgeons. There are in addition 44 hospital assistants. The Association has now 65 hospitals and dispensaries either connected with it or directly under its control. Of these 10 were constructed and are entirely supported by native chiefs and princes. The first object of the Fund in regard to these hospitals was the relief of the "Purda-nashins," and it is here that the principal difficulties have been encountered. To attract this class, it appears that the buildings must be absolutely detached from the main hospital where non-Purda women are admitted, and must be strictly closed to males, whether for inspection or other purposes. In Bengal, according to the report of Miss Bäumler, the lady doctor in charge of the Lady Dufferin Victoria Hospital in Calcutta, the prejudice is even more pronounced than in other parts. "Many high-class women consider it a disgrace to live in a hospital, and unfortunately their husbands and friends support these prejudices very strongly. Either they prevent her from coming in, or they take her away as soon as admitted. In Bengal I have observed the women are more shy and reserved and less ready to accept the full medical and surgical treatment we offer them. At the thought of only a very small operation they very often run away, and I have seen women of the best class who will not even allow a stethescope to be applied to their chest, or a small eye-dropper to come near their eyes." In other districts the progress is more encouraging. Many women travel long distances to obtain medical aid from the hospitals, and even the "Purda-nashin" women show a disposition to resort to the outdoor dispensaries, and to take advantage of the separate wards and buildings provided specially for their benefit. The number of patients treated in 1894 reached the total of 894,225, a rapid and steady increase in almost every centre, the numbers having doubled since 1891.

In the work of medical tuition the Association is able to show an equally encouraging record. As many as 224 female students are undergoing training at medical colleges and schools in Bombay, Calcutta, Madras, Lahore, and Agra, and additional classes for medical instruction have been formed at several other large centres. Of these students, 21 are Europeans, 50 are East Indians, and the remainder are Bengalis, Hindustanis, Mohammedans, Burmese, and native Christians. Nearly all are in receipt of scholarships, of which a large number are offered. Native ladies of the higher classes are slow in entering on the medical career; but some have courageously led the way, and, no doubt, in time their example will be followed. Arrangements have been recently concluded with the United Kingdom branch whereby a limited number of students who have completed their course of study at an Indian college, and have done good work under the Association, are enabled to proceed home and obtain a European diploma.

It is impossible to lay too much stress on the importance of the work of the Association in the training of midwives. Lord Wenlock, in appealing in the course of his able speech at the ninth annual meeting for additional funds to carry on this educational work, remarks, "We feel that in sending out these quasi-medical practitioners we are doing more good than might at first appear. We educate the native mind to see that the appalling atrocities committed by the native 'dhais' are not a necessity, and in the training of these women we at least, as has been graphically remarked, teach them 'what not to do.'"

The record of achievement comprised in the report is in every respect a cheering one, and that not purely from the medical standpoint. The key-note of the whole undertaking was struck by one of the native speakers, Mr. Monomohun Ghose, when he declared himself strengthened by this record of prejudice overcome and misunderstanding removed, in his firm belief "in the ultimate success of the great and unique experiment which England is trying, of engrafting a Western civilisation upon an Eastern stock."

### Holidays and Health.

[Readers of The Hospital in need of information about health resorts at home or abroad, or desirous of aid in forming holiday plans, are invited to send queries to Editor, 428, Strand, W.C. (marked "Travel" on outside of envelope), which will be answered under this section.]

I.

The annual holiday means a good deal more to the working portion of the community than merely a few weeks' enjoyment. The problem which thousands are pondering just now is how to lay in the greatest store of life and vigour in the shortest compass of time and for the least expenditure of money. For many thousands the refreshment of true country solitude forms the basis of a perfect holiday, and year by year this luxury of solitude becomes more difficult of attainment without expense. Wherever fares are cheap trippers are rampant, and the tired worker is just in a mood to be specially worried by the dust, glare, and noise of a frequented watering-place. Perfect freedom, the stillness of leafy ways, enough variety to lift the mind out of itself, pure air, cheap accommodation, and no expensive journeys, these are some of the dreams which float just now before the minds of those to whom every day brings a steady routine of work in some great city. For those who are willing to trust their own feet and live for a while the irresponsible life of a vagrant, all this may be very easily realised, and with far less real fatigue than is involved in the daily monotony of pier, parade, and sands. The requirements are one or two companions of genial disposition (one for company, two for comfort), and a small Gladstone or travelling bag, capable of being carried by the owner half a mile or so without inconvenience. Its contents should include, besides all usual necessaries, a change of walking shoes, a blouse for hot days, a book or two, of a size to slip into the pocket, and half a pound of tea. A walking dress of plain tweed or serge will prove most satisfactory. The plan of action will be somewhat as follows. A rough sketch of the tour, as regards the district to be traversed, having been formed in conclave, the walkers will probably elect to get a fair start, and travel their first stage by rail. Let us suppose that sea-air is the most important requirement and that no money for a long journey is forthcoming. A good point of departure is Canterbury (single fare 5s. 2d.), and there our travellers arrive in time to see the cathedral, engage rooms for the night, and buy materials for supper and breakfast. They will, of course, avoid hotels in town, and choose inexpensive rooms, no great distance from the station, on account of luggage. The travellers will meet with every variety of accommodation, ranging from the very good to the very bad, but a little experience confers a kind of instinct in selecting rooms, and as one night only is involved, the failures are not serious. When the halt for the night is made in a village, the inn will afford comfortable and, in most cases, inexpensive quarters. The price charged for a night's lodging in most small lodging houses and inns is from 1s. 6d. to 2s. a head, and a small additional charge may be made for preparing meals. The following morning the travellers decide on their next destination, which should not at most be more than twelve or fifteen miles distant, and may often be less. In good time the bags must be dispatched by rail or carrier as the case may be, and as half their pleasure will depend on being entirely unencumbered the walkers will do well to send on even wraps and cloaks unless the weather is very cold or threatening. It is perfectly safe to trust to the arrival of the luggage in good time the same day by either mode of despatch. Three miles an hour is a comfortable rate of walking, and during the morning some nine miles will be covered without any very strenuous exertion. Then will come a long halt for lunch, w[illegible]th which in passing through purely country regions the w[illegible]kers will have provided themselves before starting. Milk, ginger-beer, cider, and perhaps fruit they will meet at any farmhouse on the way, and lingering long at their meal under a hay stack or in some wayside copse during the noonday heat, they will taste the very ecstasy of solitude and freedom, unshackled by any obligation and supremely content in the pleasure of the moment. Another hour or two of walking will bring the travellers any time they please to their destination for the night, which having started from Canterbury may be Whitstable or Herne Bay. Here they choose rooms, fetch their bags, and lay in supplies before seeing as much of as little of the place as they desire. From this point they proceed leisurely round the coast, stopping a day or two at choice where quarters prove specially attractive or bad weather intervenes, or one of the party feels fatigued, avoiding the big towns such as Margate or Ramsgate for night stoppages, but richly enjoying their humours as they traverse them coastwise in the course of their morning's walk. In the course of a fortnight, stopping for Sundays and averaging no more than ten miles a day, the travellers, even in this leisurely way, will have reached Eastbourne or Brighton, whence they may return to London, having passed through some two dozen towns, little and big, lived all day in the purest air to be found in England, and enjoyed many hours of perfect secluded scenery entirely undisturbed by the tripper who seldom ventures more than a mile from his haunts. There is no happier way of tasting the individual charm of each region traversed; the wild freedom of the cliffs round the North Foreland and the curious peaceful beauty of Pett level can only be properly appreciated when something of the vagrant spirit, unfettered by place or hour, is superadded. No fixed itinerary has been suggested, since all depends on giving full scope to individual preference, and with a handy guide to the district the best roads and prettiest walks can be selected at will. The cost will be something as under: Railway to Canterbury and from Brighton, 9s. 8d.; carriage of luggage, 7s.; lodging (about), £1 5s.; food (2s. 6d. a day), £1 15s.; extras, 10s.

### Nurses in the U.S.A.

NEW YORK CITY TRAINING SCHOOL.

The annual report of the Training School was read by the Superintendent, Miss Louise Darche, to the large assemblage which witnessed the distribution of diplomas to thirty-one of the graduates, on the completion of their training. An address was delivered by Dr. Frederick Holme Wiggin, who, in alluding to the medical profession, is reported to have said, "We of that profession have come in the last few years to lean heavily upon you, and much of the success which has crowned our efforts has been due to the intelligent assistance of the trained nurse." In addition to diplomas and prizes a case of instruments was presented to each of the graduates.

### Nurses on the Labrador.

The two English nurses, Sister Cecilia Williams and Sister Carwardine, have commenced their third season's work amongst the fishermen and their families on the Labrador. The hospital ship of the Mission to Deep Sea Fishers made an excellent voyage, "smooth all the way," writes one of the party, "a thick fog one night and one day, and it was very cold when we got near Newfoundland, reaching St. John's harbour on June 12th." The country is in a terrible state of poverty from the bank failures, &c., and the services of the doctors and nurses will be taxed to their uttermost extent this season.

### Where to Go.

At Courtfield House, Courtfield Gardens, a sale of work will take place on Monday, July 15th, at half-past three, in aid of the Maternity Charity and District Nurses' Home, Plaistow, E.

### Notes from Germany.

By a Correspondent.

At present there are 155 sisters belonging to the "Deaconess Parent Branch Society" of the Paul Gerhardt-Stift, and they work in the districts of the province Brandenburg, and in Berlin. The Home is under the patronage of the Kaiserin, who takes a lively interest in nursing work. During the past year these sisters have tended more than 10,000 adult sick poor, as well as 2,000 children. In the same period 560 patients have been treated in the hospital attached to the home, the greater number gratuitously, and all of them irrespective of religious denomination. In the course of the spring a committee was formed in Munich which has for its object the erection of homes for the poor who suffer from diseases of the lungs and chest. Prince Ludwig, the heir apparent to the Bavarian throne, has consented to become patron to the society. Geheimrath von Lieinssen, in his speech to the newly-formed committee, observed that although much had already been accomplished in this respect on behalf of the better classes, the poorer community has been wellnigh overlooked. He recommended the formation of private societies in Bavaria, which, in course of time, might become public ones. The capital of the Munich society amounts to M32,000 (£1,600). In the first instance it is proposed to purchase a farmhouse in a healthy, mountainous district, which can be enlarged when the funds permit. Two similar homes have already been opened, and are carried on at an annual expenditure of M18,000 (£900). According to statistics, the average number of the severe cases of disease cured is 25 per cent.; of lighter cases, 60 per cent.

A folding ambulance, likely to prove of service both in the field and at home, has been constructed by an engineer, Herr Max Nehemias, of Altona. It is of simple construction, consisting of strong linen cloth and steel rods, one fitting into the other telescopically for the convenience of transport. The linen is adjusted to the framework by straps and buckles, and there is a light pillow for the head, and a pocket for the conveyance of bandages and instruments. This ambulance has secured the approval of the military authorities, who have ordered a number for military use. It was designed in the first place for the mining districts, where little has hitherto been done to cope with accidents, which are of frequent occurrence.

An interesting article on the subject of rickets has been contributed to a recent *Zeitschrift für Krankenpflege* by Professor Dr. E. Hagenbach-Burckhardt. No one who has spent any time in Germany can have failed to be struck by the great percentage of children suffering from the disease in large towns. Professor Hagenbach-Burckhardt states that the disease is unknown in China and Japan, it being most prevalent in Germany, England, Holland, France, and Northern Italy. In many of the Italian towns institutes have been opened for the reception of these children, who are collected from their homes each morning in a van. During the day they are amused, taught, fed, and cared for, and in the evening they are again conveyed to their homes. Professor Hagenbach-Burckhardt heartily approves this system, and recommends its adoption in Germany, where many benevolent institutions exist, but none are set apart for the reception and treatment of sufferers from rickets.

Influenza has been the means of keeping both doctors and chemists unusually busy in Berlin and other large towns. Though the number of persons suffering from the epidemic was considerable, the cases were not of so severe a character as in previous years. As in England, politicians were not spared, and the military suffered largely.

A petition has been signed by 700 householders of the little town of Andreasberg, situated in the Harz Mountains, protesting against the erection of a sanatorium for persons suffering from pulmonary diseases in the vicinity of the town. This is a project which the Hanseatic Society is anxious to carry out. The committee will in all probability choose a more isolated spot nearer the mountains, which will be preferable, both as regards the climate and the health of the neighbourhood.

The inhabitants of Hohegeiss have also drawn up a petition begging the Government to forbid the erection of a large hospital in their midst, which would be by them regarded as a source of infection. Therefore the proposed building, the gift of a North German Society, will probably be erected in the vicinity of Lüneberg instead.

Considerable improvements are contemplated in the interior arrangements of the hospital of the Kgl. Charité in Berlin, for which purpose a sum of M60,000 has been granted by the State. Among other things, new bedsteads and mattresses will be provided, and the ventilation of wards, corridors, and offices is to undergo a complete change.

At the instigation of a military surgeon, Dr. J. Ellbogen, of Tglan, an arrangement has been contrived by means of which the wounded may be conveyed in ease on an ambulance which can be fixed to any ordinary field waggon or cart, and which it is hoped may prove of considerable benefit to the army. The mattress is composed of strong canvass arranged on an outer framework of wood, the sides of which are in triangular form. The ambulance is thus by means of straps firmly buckled to the sides of the waggon or cart. Straps are also suspended from the top and bottom to ensure the wounded against all painful motion. The ambulance is arranged at some height above the cart, thus affording space in the conveyance for those slightly wounded. It has already been highly approved by several authorities of the military and medical staff, both in Germany and Austria.

### Notes and Queries.

### Queries.

(170) *Tracheotomy*.—Can any nurse advise me how to clean tracheotomy tubes?—*Isolation*.

(171) *Matron's Duties*.—From what book can I learn the duties which I undertake as matron of a small hospital?—*A*.

(172) *Protestant Institution*.—Can you tell me of any Protestant nursing homes for patients whose diseases render them offensive to other inmates?—*G. H. M.*

(175) *Brazil*.—Kindly tell me at what date an article on Yellow Fever appeared in The Hospital.—*Hartlepool*.

(176) *Epileptics*.—Please furnish me with the address of the Epileptic Colony in Bucks.—*M. S. B.*

(177) *Abroad*.—Where can I get particulars of hospitals abroad?—*Nurse S. A.*

(178) *India*.—To whom should I apply for information about Lady Roberts' nurses.—*Dublin*.

(179) *Corset*.—Kindly give me address of maker of the washing corset. I think the name is Herts.—*India*.

### Answers.

(170) *Tracheotomy (Isolation)*.—Read Dr. MacAdam Eccles' article of last week in the "Mirror." A physician tells us that he considers the simplest and best plan is to put silver tubes into boiling water with a lump of common washing soda. This alkaline dissolves the mucus, and leaves the tube perfectly clean. Brushes and feathers used too energetically tend to roughen and otherwise damage the tubes, which need very careful handling.

(171) *Matron (A)*.—You cannot learn such duties from books. The best plan would be to spend a few weeks with a lady occupying a similar post. You need to understand housekeeping, cooking, care of linen, &c., as well as those of a trained nurse and administrator in order to succeed. Plenty of common sense and tact are required. Do not make any rules for your subordinates hastily, but take care that all those you institute are conscientiously observed.

(172) *Protestant Institution (G. H. M.)*.—You will find these institutions named in "Burdett's Hospital Annual." If such patients are in a position to pay they will doubtless be received without trouble. Do you know "Friedenheim," near Swiss Cottage, or St. Luke's House, Osnaburgh Street? Both these take hopeless cases which are not likely to live many weeks, but they do not admit chronic cases of long standing.

(175) *Brazil (Hartlepool)*.—In The Hospital of June 30th, 1894.

(176) *Epileptics (M. S. B.)*.—The Colony is at Chalfont St. Peter, Bucks.

(177) *Abroad (Nurse S. A.)*.—In Burdett's Hospital Annual, published by the Scientific Press, 428, Strand, London.

(178) *India (Dublin)*.—See The Hospital of July 1st, 1893, p. cxxxvi.

(179) *Corset (India)*.—You mean Mr. Herts, of Wood Street, London, E.C.

### Everybody's Opinion.

[Correspondence on all subjects is invited, but we cannot in any way be responsible for the opinions expressed by our correspondents. No communications can be entertained if the name and address of the correspondent is not given, or unless one side of the paper only be written on.]

### THE ROYAL BRITISH NURSES' ASSOCIATION.

To the Editor of The Hospital.

Sir,—For some weeks past there have been statements made in a certain nursing paper, and also verbally circulated, which are calculated to mislead the members of the Royal British Nurses' Association and the public generally. I shall be, therefore, much obliged if you will allow me in your valuable paper to give another view of the questions at issue. It is the rule of the Association, as many know, that one-third of the members of the General Council and the Executive Committee retire annually in rotation, and others are elected in their place. But it must be clearly borne in mind that the balance of power, so to speak, is always the same, namely, that for every matron who retires a matron is appointed, and for every sister or nurse member the same applies. Therefore it can never be, as has been stated, that the power is falling into the hands of the medical men. Such a thing is impossible unless the nurse members willingly vote only as the doctors wish or suggest. Judging from past experience on the Executive Committee and the General Council, this is not at all likely, the nurse members hitherto invariably using their own judgment in recording their vote. It has been said that the Navy and Indian nursing services are to be unrepresented on the General Council this year, insomuch as Miss Hogg and Miss Lock, the lady superintendents of the above-named services, retire this year, having been members of the Council three years. This is in a measure correct, there being no other lady superintendents in the services, but there are "sisters" belonging to these services members of the Association, who can represent them (the services) for the time being, The rule about the retirement of members only says it is compulsory for one year, therefore next year these ladies will be eligible for re-election, and I trust they will again accept office, and represent the services for another period of three years. A good deal has been said and written from time to time of the "founders" of the Association. Would it not be better if this were made more definite, and names were given so that we might be quite clear as to whom the term "founders" apply? For we must never lose sight of the fact that without the hearty co-operation of H.R.H. Princess Christian and the medical men who have worked most untiringly in the interest of the nurses, and given hours of valuable time to attend meetings and otherwise help on the Association by every means in their power, we should never have been more than a small private community carrying no weight in the country. And to H.R.H. Princess Christian and the doctors we undoubtedly owe the granting of our charter so quickly. H.R.H. Princess Christian is not, as some would have the members believe, a president in name only. Far from it, for to my certain knowledge and all those who have sat on committees, she identifies herself personally with the details of the work of all committees, and works as hard, if not more so, than any member of the Association for its success. It is this particularly that I wish to impress on all the members of the Association who do not know this, and beg them to remember it and go to the annual meeting at Queen's Hall, July 24th, with unbiassed minds prepared to listen fairly to both sides of the questions under discussion and give their vote as their good sense dictates, and not, as some have said they mean to do, go to the meeting and shut their ears to all that may be said contrary to the opinions expressed by those they may at this present moment agree with. It is a fact proved beyond dispute that the only good way of avoiding party feeling in any association is to have compulsory retirement of a certain number of members of committees yearly—new members bringing in new ideas and new interests, and by this method each member in turn having an opportunity of serving on the committee, if he or she cares to accept office. This year many matrons and sisters in London and the provinces retire in their turn from the General Council and the Executive Committee, myself amongst the number. Is it right or fair to imply that because some are better known than others in the list of those retiring, that therefore those appointed in their stead will not do as good service to the Association in their turn as has been done by others? Let us wait and judge by results, and next year there will be vacancies again to be filled, and these members, who have done good service in the past, can again stand for re-election. In conclusion, let me remind the members of our motto, and be "steadfast and true" to the interests of our Association, of which we have good reason to be proud.—I remain, yours faithfully, L. C. East,

Lady Superintendent of the National Hospital, Queen Square, London; Member of the R.B.N.A. (late member of Executive Committee and Member of the General Council).

### Novelties for Nurses.

### A WASHING CORSET.

Hygienic clothing is as much in favour as ever, and with the increasing development of athletics among women the demand becomes greater for the articles which furnish this more convenient style of dress. The corset presents one of the greatest difficulties in the matter, and the stiff casing of bones and steels to which we have been so long accustomed having been condemned, the question arises how satisfactorily to replace it. Mr. Herts (Wood Street, E.C.) has hit on an excellent arrangement whereby the symmetry of the female form is duly preserved without the harmful pressure that results from the use of an ordinary corset. The "Health Washing Corset" is an invention that will prove a boon to thousands of growing girls and young women. Made of white coutil, it ends about a couple of inches below the waist, and the bones are inserted in such a manner as to be easily removable for washing. The busks are on the same plan, and in addition are guaranteed not to break. To nurses a corset of this description will be a godsend, as it is very comfortable, at the same time affording the necessary support to the body; it washes easily, and all bones can be replaced immediately without the intermediary of needle and cotton.

### A TOILET NECESSITY.

We have much pleasure, now that the hot weather has again set in, to call the attention of all to the refreshing results of the use of Rowland's "Kalydor." To those who have once used the lotion the Kalydor is a household word. It has an extremely pleasant effect upon the skin, and effectually prevents any roughness or chapping consequent on a long exposure to the air. To any out-door life the lotion is an invaluable accessory; and we can confidently recommend the purchase of a bottle, the pleasant effect of which more than repays the outlay on it.

### A Handsome Legacy.

Substantial appreciation of the value of district nurses for the sick poor has been shown by the late Mr. Charles Soames of Blackheath. He has left the sum of £32,000 as a perpetual fund for providing trained district nurses for the sick poor resident in their own homes in five Greenwich parishes.

### The Book World for Women and Nurses.

[We invite Correspondence, Criticism, Enquiries, and Notes on Books likely to interest Women and Nurses. Address, Editor, The Hospital (Nurses' Book World), 428, Strand, W.C.]

### HOLIDAYS AND HEALTH.

Dictionary of Mineral Waters, Climatic Health Resorts, Sea Baths and Hydropathic Establishments. With Maps, Plans, &c. B. Bradshaw. (Kegan Paul, Trench, Trubner, and Co., Limited).

The appearance at this season of manifold guides and new editions of guides sounds an annual warning note of journeys in prospect, welcomed or abhorred according to temperament. Among new editions of old public favourites one of the best is Bradshaw's "Bathing Places and Climatic Health Resorts," the general usefulness of which can hardly be overrated. Every bathing place in Europe with any degree of possibility for the English visitor is here noted, with a concise and accurate account of the properties of each mineral spring, and a comprehensive list (furnished with a good index) of the maladies for which they are likely to be of service. An excellent little table showing the quickest route, mode of conveyance, times and fares to the various health resorts, is prefixed, and is likely to be of the greatest value in helping the traveller to make his preliminary plans. It is much to be desired that one or two blemishes may in subsequent editions disappear from this useful little book. The glossary which professes to give explanations of medical terms is ineffectual to the point of absurdity. It is difficult to see how it can aid any traveller to be informed that homœopathy is "a school of medicine founded by Hahnemann," or that idiosyncrasy is "an individual peculiarity"; but the information conveyed is at any rate harmless, while such definitions as "a severe form of feverish cold" for influenza, or "disease germs in the blood" for bacteria, are not only futile but actually misleading. A more serious defect is the arbitrary selection of the names of one or two doctors for the benefit of the visitors to each resort. If the names given happen to be correct, it results in a mere species of advertisement of a highly undesirable kind, since it is by no means possible for the editor to gauge the reputation of a medical man as he can that of an hotel, and it would be a delicate and difficult task even for a competent authority to make a representative choice of practitioners from such crowded resorts as Bournemouth, Brighton, Southport, &c. But in many cases the names are not even correct, and can serve no purpose but the confusion of visitors. To quote but one case: For Malvern, which now numbers seventeen doctors in good practice, two names are given, and, of these gentlemen, one has been dead nearly five years and the other for no less than fourteen.

The Hospital Service Book. By Charles Parkhurst Baxter, M.A. (Published by Henry Frowde, Amen Corner. Price 2s.)

This new and enlarged edition of a book published first in 1892 has received a ready welcome. It is convenient in size, of good clear type, and contains short daily services, and prayers for special occasions, compiled from the Book of Common Prayer. The selection of hymns has been made with consideration and judgment, and the Rev. C. P. Baxter may be fairly congratulated on the Service Book he has prepared for ward use.

### For Reading to the Sick.

### FEAR.

**Motto.**

I am afraid of all my sorrows.—*E. M. L. G.*

**Verses.**

Poor tremblers at His rougher wind,
Why do we doubt Him so!
Who gives the storm a path, will find
The way our feet shall go;
The Lord yields nothing to our fears,
And flies from selfish cares;
But comes Himself where'er He hears
The voice of loving prayer.—*Anon.*

Fear not for I am here,
To hold thy trembling hand,
To lead thee through the coming year
On to the better land.

Yes! I am with thee now
To watch that ransomed heart,
To see how in its woe
It will perform its part.

My unforgotten child,
Have I not prayed and wept,
And through the silent night
A lonely vigil kept?
Implicitly resign
Into My care thy soul;
These hands that wounded thee,
Can they not make thee whole?

Fear not, thy Captain whispers,
The conflict may be hard,
But I am thy Deliverer,
Thy Shield and thy Reward.
—*Caroline M. Noel*

When gathering clouds around I view,
And days are dark and friends are few,
On Him I lean, Who, not in vain,
Experienced every human pain;
He sees my wants, allays my fears,
And counts and treasures up my tears.—*Grant.*

**Reading.**

Fear thou not for I am with thee: be not dismayed for I am thy God: I will strengthen thee; yea, I will help thee; yea, I will uphold thee with the right hand of My righteousness.—*Isa. xli.* 10.

"Casting all your care upon Him, for He careth for you."

There is a Divine care as well as a Divine will encircling us. There is a lawful carefulness, as well as a lawful will, allowed to us. . . . When we fail to believe in this Divine care—when we grieve and fret over things that come by God's appointment, worry ourselves about things which cannot be avoided, and are filled with a fear of disasters which may never come, then care has transgressed its limits and is busying itself in vain. Unless we believe that the world is governed by a personal God, who knows each one of us individually, is acquainted with all our trials, and must feel sympathy with us even in the least of them—unless we believe this we cannot carry out the counsel here given us by St. Peter; but as we grow in such a faith we shall grow also in the power of casting our care upon Him. How is it that faith is said to save the soul? Is it not because it anchors its hopes, its convictions, its affections in heavenly places, in a harbour over whose waters the love and stability of God have formed an atmosphere of serenity and peace?

—*H. M. Neville.*

"I the Lord thy God will hold thy right hand, saying unto thee, Fear not, I will help thee."

## Figures and Tables

**Fig. 32 f1:**